# Comparative evaluation of dynamic risk stratification according to ATA 2015 and ATA 2025 in low-risk differentiated thyroid cancer without radioiodine ablation

**DOI:** 10.1007/s12020-025-04548-6

**Published:** 2026-02-16

**Authors:** Pablo Fernández Velasco, Paula Peciña Melgosa, Beatriz Torres Torres, Luis Miguel Torres Morientes, Ana Fernández Rodríguez, Marta Alonso Mesonero, Marta de Uribe Viloria, María Álvarez Quiñones, Jaime Santos Pérez, Daniel de Luis Román, Gonzalo Diaz-Soto

**Affiliations:** 1 Centro de Investigación de Endocrinología y Nutrición Clínica (CIENC), Facultad de Medicina, Universidad de Valladolid, Valladolid, Spain; 2 Department of Endocrinology and Nutrition, Hospital Clínico Universitario de Valladolid, Valladolid, Spain; 3 Department of Otorhinolaryngology, Hospital Clínico Universitario de Valladolid, Valladolid, Spain; 4 Department of Pathology, Hospital Clínico Universitario de Valladolid, Valladolid, Spain

**Keywords:** Differentiated thyroid carcinoma, Dynamic risk stratification, ATA guidelines, Thyroglobulin, Anti-thyroglobulin antibodies, Low-risk thyroid cancer

## Abstract

**Purpuse:**

To compare dynamic risk stratification (DRS) according to the 2015 American Thyroid Association-Momesso et al. 2016 extension (ATA2015-M) and the 2025 ATA update in low-risk differentiated thyroid cancer (DTC) managed without radioactive iodine (I-131), and to explore the role of an intermediate thyroglobulin (Tg) cutoff of 1 ng/mL.

**Methods:**

We conducted a retrospective analysis of a prospectively collected cohort of 74 low-risk DTC patients treated with total thyroidectomy (n = 55) or hemithyroidectomy (n = 19) between 2020 and 2024. Clinical, histopathological, and biochemical data were collected. DRS was assessed at the first follow-up visit (6 months after surgery) and at the last visit (median follow-up 27 months [IQR 16–41]) using ATA2015-M and ATA2025 criteria. An exploratory analysis applying a Tg cutoff of 1 ng/mL was performed.

**Results:**

According to ATA2015-M, excellent response (ER) rates in total thyroidectomy patients increased from 49.2% at baseline to 52.8% at final follow-up. In contrast, ATA2025 classified 89.1% as ER at baseline and 98.2% at final follow-up (p < 0.001). Using the intermediate cutoff of 1 ng/mL, ER rates were 80.0% and 89.1%, respectively. Reclassification to ER under ATA2025 was primarily driven by anti-thyroglobulin antibody (TgAb) negativization, as Tg values remained stable and below the new 2.5 ng/mL threshold. No structural incomplete responses were observed.

**Conclusion:**

ATA2025 criteria substantially increase ER classification in low-risk DTC patients managed without I-131 compared with ATA2015-M. A 1 ng/mL Tg cutoff may provide a more realistic representation of clinical practice. The dynamic trend of TgAb, rather than their presence alone, is a key determinant for reclassification during follow-up.

## Introduction

Differentiated thyroid carcinoma (DTC) is the most frequent endocrine malignancy, and its incidence has progressively increased over the past decades, largely due to the widespread use of cervical ultrasound and other imaging techniques [1]. Despite this rise, disease-specific mortality remains low and stable, with very favorable long-term survival rates [2]. In this context, one of the main goals in the current management of DTC is to establish appropriate risk stratification for recurrence, allowing individualized therapeutic and follow-up strategies [3].

One of the tools developed for this purpose is dynamic risk stratification (DRS), proposed in 2010 and first incorporated into the American Thyroid Association (ATA) guidelines in 2015 (ATA2015) [4, 5]. DRS is based on the continuous reassessment of recurrence risk according to the response to initial therapy during follow-up. It incorporates dynamic parameters such as serum thyroglobulin (Tg) levels, trends in anti-thyroglobulin antibodies (TgAb), and imaging findings, which allow patients to be reclassified into prognostic categories of response: excellent (ER), indeterminate (IR), biochemical incomplete (BIR), or structural incomplete. Although initially conceived and validated in patients undergoing total thyroidectomy followed by radioactive iodine (I-131) ablation, in 2016 a specific extension was proposed for patients managed without ablation [6]. This aimed to broaden its applicability in an increasingly common clinical setting, particularly after the publication of the ESTIMABL2 and IoN trials in 2018 and 2025, respectively [7, 8].

Importantly, the 2016 extension of the DRS retained the biochemical thresholds for Tg and TgAb established in the 2015 ATA guidelines to define an excellent response, applying them uniformly to patients treated with I-131 and to those managed without ablation. The most recent update of the ATA guidelines in 2025 (ATA2025) [3] introduced substantial modifications in the definition of response categories for patients with DTC managed without radioactive iodine after total thyroidectomy or hemithyroidectomy. Among the most relevant changes are an increased cutoff for non-stimulated Tg (≤2.5 ng/mL) to define ER, simplification of criteria for intermediate categories, and greater emphasis on the temporal trend of TgAb rather than interpretation based solely on absolute values. To contextualize these changes, Table 1 summarizes the main differences between the ATA2015, the Momesso 2016 extension (ATA2015-M) and the updated ATA2025 criteria specifically for non-ablated patients.

**Table 1:** Dynamic risk stratification after total thyroidectomy: ATA2015 (with I-131 treatment) vs Momesso 2016 (without I-131) vs ATA2025 (without I-131)

RESPONSE CATEGORY	ATA 2015	Momesso et al. 2016	ATA 2025
EXCELLENT RESPONSE	• Negative imaging and: – Suppressed Tg < 0.2 ng/mL or – Stimulated Tg < 1 ng/mL	• Negative imaging • Non‑stimulated Tg < 0.2 ng/mL or stimulated Tg < 2 ng/mL • Undetectable TgAb	• Non‑stimulated Tg < 2.5 ng/mL
BIOCHEMICAL INCOMPLETE RESPONSE	• Negative imaging and: – Suppressed Tg ≥ 1 ng/mL or – Stimulated Tg ≥ 10 ng/mL – Rising anti‑Tg antibodies	• Negative imaging • Non‑stimulated Tg > 5 ng/mL or stimulated Tg > 10 ng/mL • Increasing Tg over time • Rising TgAb	• Non‑stimulated Tg > 5 ng/mL or • Rising TgAb (negative imaging)
STRUCTURAL INCOMPLETE RESPONSE	• Structural/functional evidence of disease • Any Tg level • With or without anti‑Tg antibodies	• Structural/functional evidence of disease regardless of Tg/TgAb	• Structural evidence of disease (local/regional/distant)
INDETERMINATE RESPONSE	• Nonspecific imaging findings or • Faint uptake in thyroid bed on RAI scan or • Detectable non‑stimulated Tg < 1 ng/mL or • Detectable stimulated Tg < 10 ng/mL or • Stable/declining TgAb without structural disease	• Nonspecific imaging findings or • Faint uptake on RAI or • Non‑stimulated Tg 0.2–5 ng/mL or • Stimulated Tg 2–10 ng/mL or • Stable/declining TgAb without structural disease	• Nonspecific imaging findings or • Non‑stimulated Tg 2.5–5 ng/mL or • Stable/declining TgAb

*ATA* American Thyroid Association

To date, no studies have evaluated the clinical impact of these modifications in DRS. Specifically, no analyses have compared patient reclassification when applying the new ATA2025 criteria versus ATA2015-M, particularly in low-risk cohorts treated exclusively with total thyroidectomy. This group represents the majority of DTC cases in current clinical practice [9, 10] highlighting the importance of assessing this issue and its potential clinical implications. Furthermore, the Tg cutoff proposed for ER in the 2025 ATA guidelines lacks robust prospective validation, differs substantially from thresholds used in prior guidelines and trials in similar cohorts, and is based mainly on expert consensus rather than strong clinical evidence.

The aim of the present study was to perform a comparative evaluation of DRS according to ATA2015-M and ATA2025 criteria in a prospective cohort of low-risk DTC patients managed without radioactive iodine ablation, and to explore the potential utility of an intermediate Tg cutoff in the definition of ER.

## Materials and methods

We conducted a retrospective analysis of a prospectively collected longitudinal cohort including all patients diagnosed with differentiated thyroid carcinoma (DTC) who underwent total thyroidectomy or hemithyroidectomy between January 2020 and December 2024 at a tertiary care thyroid unit. None of the patients received radioactive iodine ablation, and all had a minimum follow-up of 1 year from the initial surgery to the last control visit.

Clinical data were collected, including age at diagnosis, sex, disease duration, mode of diagnosis, and replacement therapy dose. Histologic data included DTC subtype and characteristics (tumor size in centimeters, multifocality, bilaterality, capsular and vascular invasion, and TNM staging). Risk of recurrence at diagnosis and DRS at 6 months after initial therapy and at the last follow-up visit were also recorded, according to ATA2025 guidelines [3, 5, 6].

Follow-up laboratory parameters were obtained using electrochemiluminescence immunoassay (ECLIA), with identical methodology applied throughout the study period (Roche Diagnostics, Geneva, Switzerland). Specifically, we collected serum TSH, anti-thyroglobulin antibodies (TgAb), and basal ultrasensitive thyroglobulin (Tg) levels under levothyroxine therapy, measured with the second-generation Elecsys® Tg assay on the Cobas® e801 platform, following the manufacturer’s instructions (Roche Diagnostics, Geneva, Switzerland).

All patients provided written informed consent prior to inclusion. The study protocol was approved by the Institutional Clinical Research Ethics Committee (PI 21-2404 and PI-24-460-C) and was conducted in accordance with the Declaration of Helsinki.

## Statistical analysis

The sample size was calculated for a paired comparison of correlated proportions, assuming that 30% of patients would be reclassified from non-excellent to excellent response. A statistical power of 85% (β = 0.15) and a significance level of 0.05 (α = 0.05) were used. Based on these parameters, a minimum of 51 paired observations was required.

Normality of the variables was assessed using the Kolmogorov–Smirnov test. Quantitative variables showing a normal distribution were reported as mean and standard deviation, while those not meeting normality criteria were reported as median and interquartile range [IQR]. Qualitative variables were presented as absolute frequencies and percentages. For comparisons of paired categorical data (specifically, comparisons among the different DRS—ATA 2015/Momesso et al., ATA 2025, and the intermediate Tg cutoff of 1 ng/mL), McNemar’s test or the Stuart–Maxwell test was used, as appropriate. Agreement between classification systems was assessed with Cohen’s kappa coefficient, interpreted according to Landis and Koch criteria.

Paired continuous variables were compared using the paired Student’s t test or, in case of non-normal distribution, the Wilcoxon signed-rank test. Correlations between continuous variables were analyzed using Pearson’s correlation coefficient. Logistic regression analyses were performed to identify predictors of elevated basal Tg at three predefined cutoffs (Tg >0.2 ng/mL, Tg >1 ng/mL, and Tg >2.5 ng/mL). For each cutoff, an independent logistic model was constructed. The following covariates were included in all models based on clinical relevance: age at diagnosis, sex, tumor size, multifocality, bilaterality (for total thyroidectomy patients), capsular invasion, histological subtype, and TSH level at the initial visit.

A p value < 0.05 was considered statistically significant. All analyses were conducted using IBM SPSS Statistics version 23.0 (IBM Corp., Armonk, NY, USA) and RStudio v2025.06.0 (Boston, MA, USA).

## Results

Seventy-four patients met the inclusion criteria during the study period. Of these, 82.4% were women, and the mean age at diagnosis was 53.9 ± 11.6 years, with a median follow-up 27 months [IQR 16–41].

A total of 74.3% underwent total thyroidectomy and 25.7% hemithyroidectomy. In 66.2%, the diagnosis was incidental after surgery for another condition. Histologic evaluation showed that papillary carcinoma was the predominant subtype (47.3% classic variant, 39.2% follicular variant, and 10.8% oncocytic variant), followed by follicular carcinoma (1.4%) and oncocytic follicular carcinoma (1.4%). The mean primary tumor size was 7.5 ± 5.1 mm. Multifocality was present in 35.1% of patients, with bilateral involvement in 32.7% of total thyroidectomies. Capsular invasion was documented in 16.2% of cases, without evidence of vascular invasion, extrathyroidal extension, lymph node involvement, or distant metastases. All patients were classified as stage I (AJCC, 8th edition) and 64.9% as low risk of recurrence (ATA2025). Clinical, histopathologic, and postoperative findings are summarized in Table 2.

**Table 2:** Clinical, Histopathological, and Initial Postoperative Findings

Variable	Overall Cohort (n = 74)	Total Thyroidectomy (n = 55)
**Baseline clinical and histopathological characteristics**		
Women, n (%)	61 (82.4%)	45 (81.8%)
Age at diagnosis (years, mean ± SD)	53.9 ± 11.6	56.55 ± 11.45
Follow-up duration (months, median [IQR])	27 [16–41]	26 [16–41]
Patients on levothyroxine therapy, n (%)	64 (86.5%)	–
Levothyroxine dose (μg/day, mean ± SD)	114.8 ± 40.7	–
Type of surgery, n (%)	Total thyroidectomy: 55 (74.3%) Hemithyroidectomy: 19 (25.7%)	–
Incidental diagnosis, n (%)	49 (66.2)	34 (63.0%)
Histological subtype, n (%)	Papillary, classic variant: 35 (47.3%) Papillary, follicular variant: 29 (39.2%) Papillary, oncocytic variant: 8 (10.8%) Follicular carcinoma: 1 (1.4%) Oncocytic carcinoma: 1 (1.4%)	Papillary, classic variant: 25 (45.5%) Papillary, follicular variant: 24 (43.6%) Papillary, oncocytic variant: 6 (10.9%)
Primary tumor size (mm, mean ± SD)	7.5 ± 5.1	8.48 ± 4.40
Multifocality, n (%)	26 (35.1%);	20 (36.4%) Bilateral: 18 (32.7)
Capsular invasion, n (%)	12 (16.2%)	8 (14.5%)
Vascular, extrathyroidal, nodal extension, or metastasis	Absent	Absent
AJCC stage (8th edition), n (%)	I: 74 (100)	I: 55 (100)
ATA2025 risk category, n (%)	Low risk recurrence: 48 (64.9) Low-intermediate risk recurrence: 26 (35.1)	Low risk recurrence: 29 (52.7) Low-intermediate risk recurrence: 26 (47.3)
**Initial postoperative visit findings (~6 months)**		
Initial TSH (μIU/mL, median [IQR])	1.53 [0.3 – 4.73]	1.28 [0.3 – 5.54]
Basal thyroglobulin (ng/mL, median [IQR])	0.355 [0.093 – 1.403]	0.23 [0.04–0.49]
Anti-thyroglobulin antibodies (IU/mL, median [IQR])	18.0 [15.0 – 35.1]	18.0 [15.0–32.4]
Positive TgAb, n (%)	9 (12.2)	4 (7.2)
Initial cervical ultrasound	No structural abnormalities	No structural abnormalities

At the initial follow-up visit, median TSH was 1.53 [0.3 – 4.73] μIU/mL, median thyroglobulin (Tg) was 0.355 ng/mL [0.093–1.403], and median anti-Tg antibody levels were 18.0 [15.0–35.1] IU/mL. No structural abnormalities were identified on cervical ultrasound. According to ATA2015-M DRS, the initial response was categorized as excellent in 51.4%, indeterminate in 45.9%, and biochemical incomplete in 2.7%. At the final follow-up visit, these proportions were 55.4%, 43.2%, and 1.4%, respectively (p < 0.05).

**Fig. 1:**
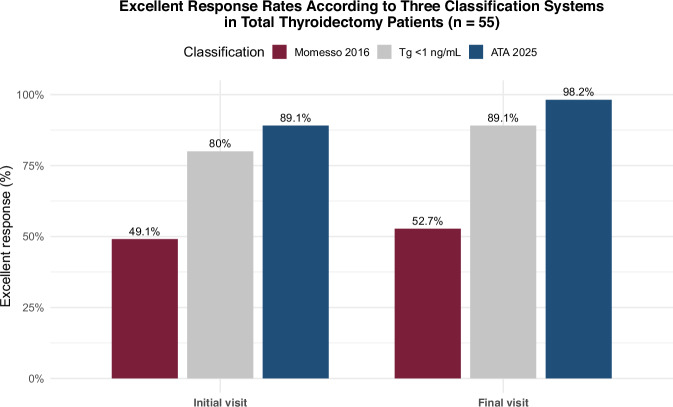
Excellent Response Rates According to Three Classification Systems in Total Thyroidectomy Patients (n = 55)

**Fig. 2:**
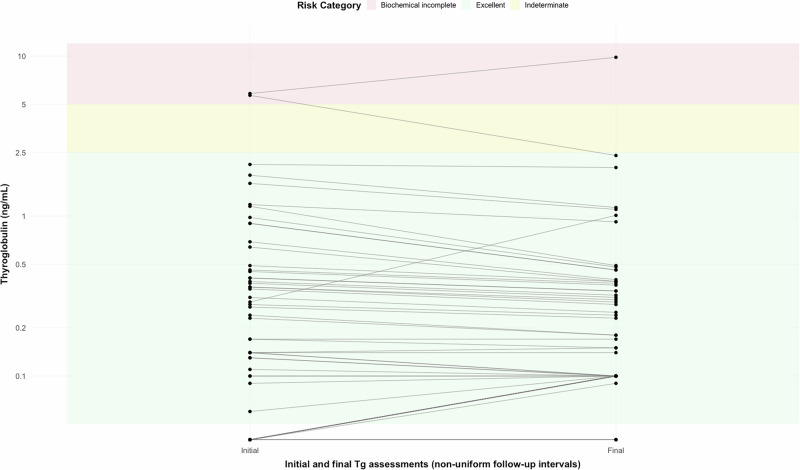
Distribution of Basal Thyroglobulin Levels at Initial and Final Follow-up visit and Dynamic Risk Stratification According to ATA 2025 in Total Thyroidectomy Patients (Thyroglobulin in logarithmic Scale)

In the subgroup with total thyroidectomy (n = 55), ATA2015-M initially classified 49.2% as excellent response, 47.6% as indeterminate, and 3.6% as biochemical incomplete. At the final visit, the distribution was 52.8%, 45.4%, and 1.8%, respectively (p < 0.05). In contrast, ATA2025 categorized 89.1% as excellent response at baseline and 98.2% at the final assessment (p < 0.01) (Table 3 and Fig. 1). Figure 2 illustrates the individual evolution of Tg levels from the initial to the final follow-up visit according to ATA2025 (Fig. 2).

**Table 3:** Evolution of Dynamic Risk Stratification and Biochemical Markers During Follow-up

Classification	Initial	Final	p-value
**ENTIRE COHORT, n** = **74**			
**Momesso 2016 DRS (Entire cohort)**			<0.05
Excellent response n (%)	38 (51.4)	41 (55.4)	—
Indeterminate response n (%)	34 (45.9)	32 (43.2)	—
Biochemical incomplete n (%)	2 (2.7)	1 (1.4)	—
**TOTAL THYROIDECTOMY, n** = **55**			
**Momesso 2016 DRS (Total thyroidectomy)**			<0.05
Excellent response n (%)	27 (49.2)	29 (52.8)	—
Indeterminate response n (%)	26 (47.6)	25 (45.4)	—
Biochemical incomplete n (%)	2 (3.6)	1 (1.8)	—
**ATA 2025 DRS (Total thyroidectomy)**			<0.01
Excellent response n (%)	49 (89.1)	54 (98.2)	
Indeterminate response n (%)	4 (7.2) (4 TgAb + )	0 (0)	—
Biochemical incomplete n (%)	2 (3.6)	1 (1.8)	—
**Tg Cutoff** < **1 ng/mL (Total thyroidectomy)**			<0.001
<1 ng/mL, n (%)	44 (80.0)	49 (89.1)	—
1–2.5 ng/mL and/or TgAb positive, n (%)	9 (16.4) [5 Tg / 4 TgAb + ]	5 (9.1) [5 Tg / 0 TgAb + ]	—
>2.5 ng/mL, n (%)	2 (3.6)	1 (1.8)	—
**Biochemical Markers (Entire cohort, n** = **74)**			
Tg (ng/mL), median [IQR]	0.355 [0.093–1.403]	0.295 [0.100–1.123]	0.457
TgAb (IU/mL), median [IQR]	18.0 [15.0–35.1]	18.5 [16.0–25.5]	0.117
TgAb positive, n (%)	9 (12.2)	4 (5.4)	0.001

*TgAb+* Thyroglobulin antibody–positive status; *Tg* Thyroglobulin levels

When applying an intermediate cutoff between ATA2015-M and ATA2025 of Tg = 1 ng/mL, 80% of patients at baseline and 89.1% at the final visit presented values below 1 ng/mL (p < 0.001) (Table 3 and Fig. 1). Comparison of the 1 ng/mL cutoff with ATA2015-M and ATA2025 showed significant differences (p < 0.001 and p < 0.05, respectively), favoring a higher identification of excellent response with ATA2025. Overall concordance between the classifications was low for ATA2015-M et al vs ATA2025 (κ = 0.185) and ATA2015-M et al vs intermediate cutoff (κ = 0.352), and moderate for intermediate cutoff vs ATA2025 (κ = 0.518).

Regarding the etiology of non-excellent responses, no cases of structural incomplete response were observed. At baseline, ATA2015-M et al attributed most non-excellent responses to elevated Tg levels (86.7%) and a smaller proportion to positive TgAb (13.3%), categorized as IR. In contrast, ATA2025 showed the opposite pattern, with 66.7% of non-excellent responses due to positive TgAb and 33.3% to elevated Tg levels (p < 0.005).

Tg levels remained stable during follow-up (0.355 [0.093–1.403] vs 0.295 [0.100–1.123] ng/mL; p = 0.457), with a strong correlation between baseline and final values (r = 0.951; p < 0.001). TgAb levels also remained stable (18.0 [15.0–35.1] vs 18.5 [16.0–25.5] IU/mL; p = 0.117), although the proportion of TgAb-positive patients significantly decreased from 12.2% to 5.4% (p < 0.001). Thus, the greater reclassification to excellent response according to ATA2025 was primarily driven by TgAb negativization, since Tg values remained stable and below the recently established cutoff (Table 3).

In the logistic regression analyses, no significant predictors of elevated basal Tg were identified for any of the evaluated cutoffs. In the model using Tg >1 ng/mL, neither tumor size (OR = 0.88; p = 0.09) nor multifocality (OR = 3.08; p = 0.11) showed statistically significant associations. Similarly, no significant associations were observed in the models using the 0.2 ng/mL or 2.5 ng/mL thresholds.

## Discussion

Clinical practice guidelines for DTC have progressively evolved toward less aggressive strategies, prioritizing individualization based on recurrence risk throughout follow-up. In this context, the incorporation of DRS into the ATA 2015 guidelines represented a relevant conceptual shift, allowing continuous reassessment of prognosis [3–5] The 2025 ATA update introduces essential changes in the definition of DRS response categories for patients managed without I-131ablation.

In our cohort of 74 low-risk DTC patients treated exclusively with surgery, applying the updated DRS criteria (ATA 2025) markedly increased the proportion of patients classified as having an excellent response. In the subgroup with total thyroidectomy, ER increased from 49.2% at baseline using ATA2015-M to 89.1% using ATA2025, and from 52.8% to 98.2% at the final follow-up visit. These findings are comparable to those reported in similar cohorts prior to the ATA 2025 update [11, 12]. The more than one-third increase in ER classification according to ATA 2025 was mainly attributable to the substantial rise in the basal Tg threshold, which is markedly higher than the previously proposed value (0.2 vs 2.5 ng/mL). Furthermore, the present study demonstrates a change in the pattern of classification among patients with non-excellent responses. Whereas under ATA2015-M most cases were attributed to elevated Tg levels ( ≥ 0.2 ng/mL; 86.7%), a smaller proportion were related to positive TgAb (13.3%). With the higher Tg threshold introduced by ATA2025, TgAb positivity becomes the predominant cause (66.7%), while only 33.3% of non-excellent responses are attributable to elevated Tg levels (p < 0.005). This modification not only influences classification but also impacts follow-up strategies and decisions regarding adjuvant therapy, including salvage I-131 treatment.

Critical analysis of the thresholds underlying both definitions of ER reveals certain limitations. First, ATA 2015-M applied the same non-stimulated Tg cutoff (0.2 ng/mL) to patients managed exclusively with thyroidectomy as to those treated with surgery plus I-131 ablation, despite marked differences in thyroid remnant volume and Tg kinetics between these two scenarios [13] Indeed, for the same non-stimulated Tg level, the likelihood of structural disease is higher in patients treated with I-131 compared with those treated exclusively with surgery [6, 14, 15].

By contrast, ATA2025 defines ER as basal Tg ≤2.5 ng/mL, representing an increase of +1150% relative to the prior 0.2 ng/mL threshold. This change was introduced empirically, without strong supporting evidence, as acknowledged in the guideline itself. Although the higher cutoff increases the proportion of ER in a subgroup of low-risk DTC patients, it may also lead to overclassification and reduced sensitivity to detect clinically relevant disease [7, 8]. In addition, several studies in cohorts similar to ours have proposed intermediate cutoffs between 0.2 and 2.5 ng/mL for non-stimulated Tg as criteria for ER, which could represent a more conservative alternative while still markedly increasing ER rates [7, 8, 11, 13, 16].

In our series, using an intermediate Tg cutoff of 1 ng/mL classified as ER 80.0% of patients at baseline and 89.1% at the final visit. This results differed significantly from ATA2015-M (p < 0.001) and ATA2025 (p < 0.05), showing moderate concordance with ATA2025 and weak-to-moderate concordance with ATA2015-M. Importantly, the 1 ng/mL cutoff aligns with the value used in the ESTIMABL2 trial [7]—the largest prospective study in low-risk non-ablated DTC—supporting its clinical relevance.

Further insight emerges when evaluating Tg cutoffs specifically in total thyroidectomy patients. At baseline, raising the threshold from 0.2 to 1 ng/mL increased the ER rate from 49.2% to 80% ( + 30.8 points; +63% relative). Increasing the cutoff further to 2.5 ng/mL added only 9.1% more (80% to 89.1%). At the final visit, the same pattern was observed: increasing from 0.2 to 1 ng/mL raised the rate from 52.8% to 89.1% ( + 36.3 points; +69% relative), while the change from 1 to 2.5 ng/mL added only 9.1% (89.1% to 98.2%). This pattern reflects logarithmic growth, where the greatest changes occur at low Tg levels (<1 ng/mL), and subsequent increases in the cutoff have diminishing impact. Consequently, a 1 ng/mL threshold appears to better reflect the real distribution of patients in clinical practice compared with the 0.2–2.5 ng/mL interval, which groups highly heterogeneous values under the same ER category. These findings are consistent with a prospective study with a median follow-up of more than five years, in which 95% of total thyroidectomy patients without I-131 ablation and with basal ultrasensitive Tg <1 ng/mL remained free of structural recurrence [17].

Another striking observation is that ATA2025 continues to rely solely on static Tg thresholds to define DRS categories, without considering dynamic behavior, even though stable Tg levels >2.5 ng/mL may represent a lower recurrence risk than Tg <2.5 ng/mL with short doubling times [18–20].

In our cohort, no structural incomplete responses were identified; thus, all non-excellent responses were exclusively defined by biochemical levels. Comparison of ATA2015-M and ATA2025 classifications revealed a significant shift in the etiology of these responses: under ATA2015-M, most were attributed to elevated Tg (86.7%) and only a minority to positive TgAb (13.3%), whereas under ATA 2025, 66.7% were explained by TgAb positivity and only 33.3% by elevated Tg (p < 0.005). This greater relative weight of TgAb is not due to an explicit emphasis in the new classification but rather an indirect consequence of raising the Tg cutoff above the mean levels observed in high-volume surgical centers.

In patients treated with total thyroidectomy and I-131 ablation, isolated TgAb positivity does not determine prognosis; instead, the trend over time is critical [21, 22]. Similarly, recent studies in low-risk DTC patients without I-131 ablation [12, 23–25] show that isolated TgAb positivity is not associated with higher recurrence risk, but rather the temporal trend (declining vs stable/increasing) is the true prognostic marker. Considering TgAb as a static parameter overclassifies some patients as non-excellent responders, even though they eventually achieve ER during follow-up, as also seen in our cohort. Indeed, the proportion of TgAb-positive patients significantly decreased from 12.2% to 5.4% during follow-up (p < 0.001). Consequently, the majority of reclassification to ER using ATA2025 was driven by TgAb negativization, as Tg values remained stable and below the recently established threshold throughout follow-up (0.355 [0.093–1.403] vs 0.295 [0.100–1.123] ng/mL; p = 0.457), with very strong correlation between baseline and final measurements (r = 0.951; p < 0.001). This stability of Tg values at low levels has been previously described [7, 8, 16, 26, 27].

Finally, our analysis did not identify significant predictors of elevated basal Tg at any of the evaluated cutoffs. However, non-significant patterns were observed suggesting a potential inverse relationship with tumor size and a positive relationship with multifocality. These findings should be interpreted cautiously, as they did not reach statistical significance. Further investigation is warranted.

This study has several strengths. First, it is based on a cohort with prospectively collected biochemical and imaging data, ensuring homogeneous and standardized follow-up assessments in patients treated exclusively with surgery—an increasingly common but still underrepresented clinical scenario. Second, all patients were evaluated using the same ultrasensitive Tg and TgAb methodology, ensuring homogeneous determinations throughout follow-up. However, some limitations must be acknowledged. The relatively small sample size and single-center design limit the generalizability of findings. The time interval between the initial visit assessment and the last follow-up visit was not homogeneous across patients, reflecting the variability of real-world clinical follow-up. In addition, although the median follow-up of 27 months allowed evaluation of biochemical evolution and changes in dynamic risk stratification, this duration is insufficient to assess long-term structural recurrence. Finally, the absence of structural incomplete responses, although consistent with the low aggressiveness of low-risk DTC, prevents evaluation of the sensitivity of the new indeterminate and biochemical incomplete response thresholds for detecting recurrence.

In summary, applying the ATA2025 criteria markedly increases the proportion of excellent responses in low-risk DTC patients treated with surgery alone, largely due to the substantial rise in the Tg threshold. However, this reclassification is driven mainly by TgAb negativization rather than by meaningful biochemical changes, and it markedly reduces the discriminative capacity of the DRS. An intermediate Tg cutoff of 1 ng/mL may better reflect the natural distribution of basal Tg in non-ablated patients. These findings highlight the need for prospective validation of the ATA2025 criteria and for incorporating dynamic parameters, such as Tg kinetics, into future refinements of the DRS framework.

## Data Availability

No datasets were generated or analysed during the current study.
